# A transgenic approach to control hemipteran insects by expressing insecticidal genes under phloem-specific promoters

**DOI:** 10.1038/srep34706

**Published:** 2016-10-06

**Authors:** Shaista Javaid, Imran Amin, Georg Jander, Zahid Mukhtar, Nasir A. Saeed, Shahid Mansoor

**Affiliations:** 1 Agricultural Biotechnology Division, National Institute for Biotechnology and Genetic Engineering (NIBGE), P. O. Box 577, Jhang Road, Faisalabad, Pakistan; 2 Pakistan Institute of Engineering and Applied Sciences (PIEAS), Nilore, Islamabad, Pakistan; 3 Boyce Thompson Institute for Plant Research, 533 Tower Road, Ithaca, NY 14853, USA

## Abstract

The first generation transgenic crops used strong constitutive promoters for transgene expression. However, tissue-specific expression is desirable for more precise targeting of transgenes. Moreover, piercing/sucking insects, which are generally resistant to insecticidal *Bacillus thuringiensis* (*Bt*) proteins, have emerged as a major pests since the introduction of transgenic crops expressing these toxins. Phloem-specific promoters isolated from *Banana bunchy top virus* (BBTV) were used for the expression of two insecticidal proteins, *Hadronyche versuta* (Blue Mountains funnel-web spider) neurotoxin (Hvt) and onion leaf lectin, in tobacco (*Nicotiana tabacum*). Here we demonstrate that transgenic plants expressing Hvt alone or in combination with onion leaf lectin are resistant to *Phenacoccus solenopsis* (cotton mealybug), *Myzus persicae* (green peach aphids) and *Bemisia tabaci* (silver leaf whitefly). The expression of both proteins under different phloem-specific promoters resulted in close to 100% mortality and provided more rapid protection than Hvt alone. Our results suggest the employment of the Hvt and onion leaf lectin transgenic constructs at the commercial level will reduce the use of chemical pesticides for control of hemipteran insect pests.

Chemical pesticides have been used world-wide at a large scale for several decades to control insect pests^1^. Many of these insecticides not only pollute the environment, but also affect other non-target insects and vertebrates, including humans^2^. Additionally, chemical pesticides have a high potential to leach from the site of application to neighboring terrain or into underground water supplies^3^. Plants depend on animals for 75% of their pollination^4^, and 80% of this pollination is carried out by insects, in particular bees^5^. Agricultural insecticides show enormous direct and indirect impacts on non-target beneficial insects including changes in the biochemical pathways^6^,^7^, development^8^,^9^, adult longevity^10^ and fecundity^11^. Behavioral modifications have also been observed in beneficial insects. They tend to have navigation problems and cannot perform their functions in a proper manner when pesticides affect their nervous systems^11^,^12^,^13^. It has also been reported that the feeding behavior of insects is changed by either the repellent action of pesticides, antifeedant properties, or inability to locate food due to reduced olfactory capacity after exposure to the pesticides^14^,^15^,^16^. Although, only 2% of pesticides were found to affect vertebrates at agriculturally relevant concentrations, they nevertheless can have developmental, survival, genotoxic, cytotoxic, immunotoxic, and neurobehavioural effects^17^,^18^. These effects can be direct (*e.g*. exposure during application)^19^,^20^ or indirect (exposure through the consumption of food products exposed to the pesticides)^21^,^22^,^23^.

Given the profound environmental damage that can be caused by chemical pesticides, there is a clear need to develop new strategies, both to cope with highly resistant insect species and to avoid the secondary effects caused by agrochemicals. One approach is to produce transgenic crops expressing insecticidal toxins, such as genetically engineered potato, corn, and cotton expressing δ-endotoxins from the soil bacterium *Bacillus thuringiensis* (*Bt* toxins)^24^. This bacterium, or its encoded *Bt* toxin, is the globally most commonly used biopesticide^25^. Due to the extensive use of *Bt* genes in many crops, several insect species have developed resistance^26^. Nevertheless, this technology has already exceeded the predicted time span that typically passes in the field before resistance emerges to most conventional neurotoxic pesticides^26^,^27^,^28^. A variety of long-term strategies are being developed to avoid the development of insect resistance to *Bt* toxins. One successful approach is to pyramid the expression of several *Cry* genes, which encode the production of different *Bt* toxins, in crop plants^29^,^30^.

Although constitutively expressed *Bt* genes have been very successful, in some cases tissue-specific expression is a better option, for example in epidermal cells, which first come under attack from insects, or in the phloem for sap-sucking insects^31^. Hemipteran insects cause crop damage by taking up phloem sap and also serve as vectors for more than 200 plant viruses^20^. As many hemipteran insects feed exclusively from the phloem, effective resistance to these insects can be limited to the phloem cells by expressing genes under phloem-specific promoters. Using this approach, the expression of resistance genes in non-target parts and tissues can be avoided, thereby reducing the metabolic load on the transgenic plants. Implementation of this approach necessitates the identification and characterization of efficient phloem-specific promoters.

Over the past few years, several phloem-specific promoters have been utilized to develop genetically superior plants^32^. Two such promoters are *RSs1* (isolated from rice) and *rolC* (isolated from *Agrobacterium rhizogenes* strain A4). *RSs1* has already been used to regulate the expression of *β-glucuronidase* (GUS)^33^, *Galanthus nivalis* agglutinin (GNA)^34^,^35^, and *Allium sativum* leaf agglutinin (ASAL)^36^ in a phloem-specific manner. The *rolC* promoter has been used for the phloem-specific expression of the *GUS* gene in transgenic tobacco^37^,^38^ and rice^39^.

*Banana bunchy top virus* BBTV, the type member of the genus *Babuvirus* in the family *Nanoviridae*, has a phloem-limited nature^40^. This virus, which is transmitted by the green peach aphid (*Myzus persicae*), has a multipartite genome consisting of six circular ssDNAs (each ~1.1 kb), individually encapsulated within separate icosahedral virions (each ~18–20 nm in diameter). Each of the six *Babuvirus* genome components encodes a different protein in the sense strand of the virus. These include a rolling-circle replication initiator protein, Rep (encoded on DNA-R), a protein with unknown function (encoded on DNA-U3), a capsid protein (CP; encoded on DNA-S), a movement protein (MP; encoded on DNA-M), a cell-cycle-link protein (Clink; encoded on DNA-C) and a nuclear shuttle protein (NSP; encoded on DNA-N)^41^,^42^,^43^,^44^. The intergenic regions of the nuclear shuttle protein and coat protein coding genes serve as promoters and can be used for expression in plant phloem cells^45^,^46^.

Scientists searching for new insecticidal proteins and neurotoxins are attracted by the molecular and chemical diversity of spiders^47^. The most important constituents of many spider venoms are 4–10 kDa ligand peptides that are tightly folded by means of intramolecular disulfide bridges, and which include a great diversity of antagonists acting on membrane ion channels^48^,^49^. Some of these peptides have the ability to block ion channels at the neuronal level. One such example is ω-atracotoxin^50^, a specific antagonist of insect calcium channels that was recently isolated from Australian funnel web spiders by screening their venom for activity against cotton bollworms (*Helicoverpa armigera*)^51^,^52^ (see supplementary data Figure S5 for mode of action). Hvt, when expressed from a bacterial expression system, was topically toxic to insects. In feeding experiments with the chewing herbivores *H. armigera* and *Spodoptera littoralis* (Egyptian cotton leafworm), insects on transgenic plants expressing Hvt from the 35S promoter were dead within 24 h^53^,^54^,^55^,^56^.

Lectins can be found in all kingdoms of life, ranging from viruses to bacteria and animals. The classical plant lectins are often found at high concentrations in certain plant tissues (*e.g*. seeds, bark, and bulbs). However, the precise function of these proteins is yet to be elucidated. High concentrations in source tissues suggest that lectins also serve as storage proteins. Additionally lectins can serve as defensive molecules against insect herbivores and pathogens^57^. The first plant lectin shown to be active against Hemiptera was snowdrop lectin (*Galanthus nivalis* agglutinin; GNA), which is one of the most extensively studied plant lectins and is easy to purify for investigating the mode of action^33^,^58^,^59^. The most likely mechanism for the entomotoxic activity involves the interaction of lectin with different glycoproteins and glycan moieties of insect gut. Lectin binding to likely targets, numerous enzymes and glycans in the insect body, can interfere with different physiological processes^59^. Under normal conditions, when insects take up plant lectins during feeding, the first proteins that come into contact with lectins will thus be located in the digestive tract. Upon binding, the lectin is able to pass through the gut epithelium barrier^60^. GNA has been shown to bind to the midgut epithelium of *Nilaparwata lugens* (brown plant hopper) and cause disruptive morphological changes^59^ (see supplementary data Figures S3 and S4).

Cotton, the main cash crop of Pakistan, is threatened by a variety of sucking pests, including whiteflies, aphids, jassids, thrips and mealybugs. Our longer-term goal is to develop effective strategies for controlling phloem-feeding insects by gene pyramiding, as well as tissue-specific expression of insecticidal genes in cotton. Here we show that the expression of *Hadronyche versuta* (Blue Mountains funnel-web spider) ω-atracotoxin (Hvt) and onion (*Allium cepa*) leaf lectin from phloem-specific promoters provides protection against three species of phloem-feeding insects, *Phenacoccus solenopsis* (cotton mealybug), *M. persicae*, and *Bemisia tabaci* (silver leaf whitefly).

## Results

### Cloning and GUS expression assay

We successfully amplified the BBTV nuclear shuttle protein (NSP) promoter (352 bp), coat protein (CP) promoter (540 bp), Hvt (117 bp) and onion leaf lectin (333 bp). Constructs were cloned and sequenced. Homology of all of the sequences was checked by performing BLAST comparisons. The Hvt gene sequence showed 100% identity to *H. versuta* spider neurotoxin^54^ and the lectin gene sequence showed 100% identity to the *A. cepa* mannose-binding insecticidal leaf lectin^61^.

**Figure 1:**
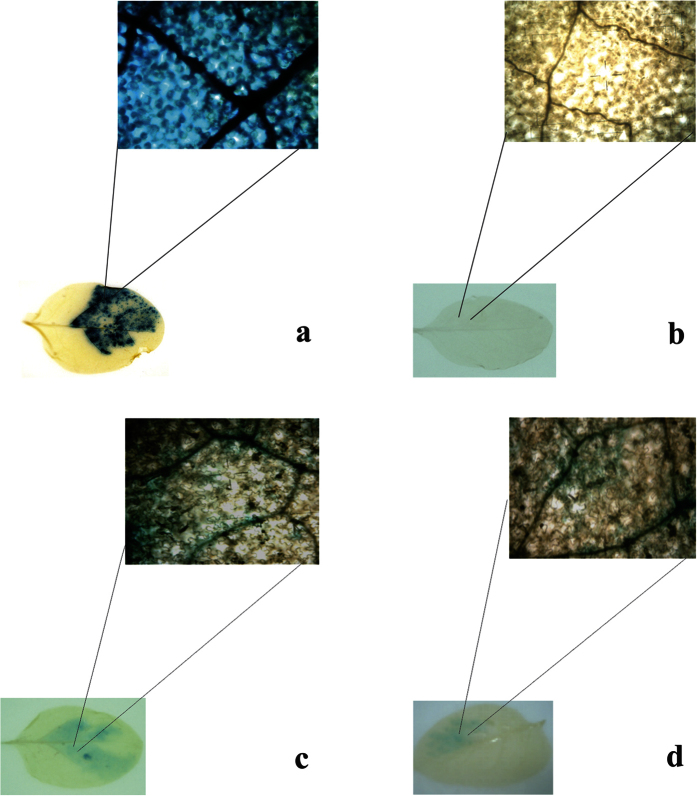
GUS-histochemical staining assay for BBTV promoter expression (**a**) Positive control 2X cauliflower mosaic virus 35S promoter (**b**) Negative control pGreen 0029 (**c**) BBTV nuclear shuttle protein (NSP) promoter (**d**) BBTV capsid protein (CP) promoter.

**Figure 2:**
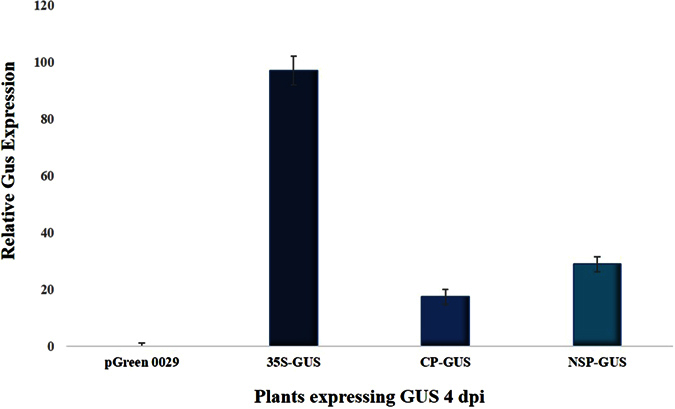
Quantification of GUS expression in *Nicotiana tabacum* using ImageJ 1.50. The four bar graphs show: pGreen0029 (−ve control), GUS expression from the empty vector; 35S-GUS (+ve control), GUS expression under 35S promoter; CP-GUS, expression of GUS under BBTV-Coat protein promoter; and NSP-GUS, GUS expression under BBTV-Nuclear shuttle protein promoter. Each bar represents the mean +/− standard error of three measurements; dpi = days post infection.

*Nicotiana tabacum* leaves were infiltrated with GUS constructs to test promoter function. The *Cauliflower mosaic virus* 35S promoter was used as a positive control and empty-vector, pGreen0029, as negative control, to assess the BBTV-promoter expression. As the 35S promoter is a constitutive promoter, it showed GUS expression in whole inoculated patch (Fig. 1a). In contrast, the pGreen0029 (−ve control) did not show GUS gene expression (Fig. 1b). Both BBTV promoters, NSP and CP, showed GUS expression only in the vascular cells (Fig. 1c,d), but expression from the NSP promoter was stronger than that from the CP promoter (Fig. 2). These results indicate that expression of GUS from both CP and NSP promoters is phloem-limited, making these promotors suitable for phloem-specific expression of Hvt and onion leaf lectin insecticidal transgenes.

### Transgene Analysis

**Figure 3:**
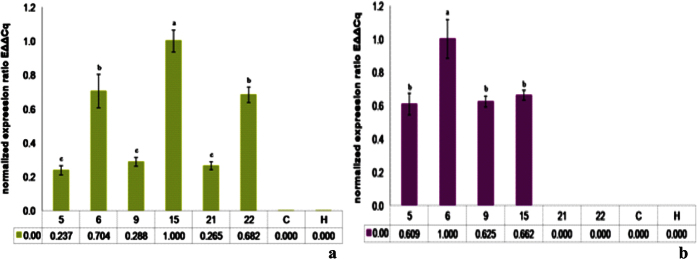
qPCR analysis for toxin gene expression in transgenic *Nicotiana tabacum* (**a**) Hvt gene expression (**b**) Lectin gene expression. Different letters above the bars indicate P < 0.01, ANOVA followed by Tukey’s HSD test. Each bar represents the mean +/− standard deviation of five plants for each line.

Transgenic tobacco plants were confirmed to carry Hvt and lectin genes at the T_1_ stage by PCR. PCR-positive plants were further processed for the extraction of RNA and cDNA synthesis. Quantitative reverse transcriptase-PCR (qPCR) showed a single peak in each of the melt curves for the genes of interest, Hvt and lectin, indicating that there was no non-specific amplification (see Supplementary data Figure S6). To allow later comparison to insect resistance traits, relative transgene expression levels were quantified in six independent transgenic lines, two single gene HVT constructs and four double gene constructs (DGC) with both Hvt and lectin (Fig. 3). In case of the Hvt gene, all of the plants showed good expression. DGC 15 was the most highly expressing line (relative expression = 1.00), expressing Hvt almost two-fold higher than the H 22 (0.682). DGC 6 is the second most highly expressing line (0.704), while in case of DGC 5, 9 and H 21 the gene was expressed at a very low level and relative expression was almost the same (0.234, 0.288 and 0.265 respectively) (Fig. 3a).

The results obtained from lectin qPCR are distinct from the Hvt results. In case of lectin, the highest level of expression was observed in DGC 6 (relative expression = 1.000). The lectin expression in DGC 6 was almost 2-fold higher than in the rest of the lines DGC 5, 9 and 15 (i.e. 0.609, 0.666 and 0.662 respectively) (Fig. 3b). The relatively high expression level was predicted to make the DGC 6 construct more toxic towards insect pests.

### Insect Bioassays

Bioassays were performed in order to assess the effectiveness of the toxin proteins against *P. solenopsis, M. persicae*, and *B. tabaci*, all of which are not only major threats to cotton but also feed on tobacco.

#### Phenacoccus solenopsis

**Figure 4:**
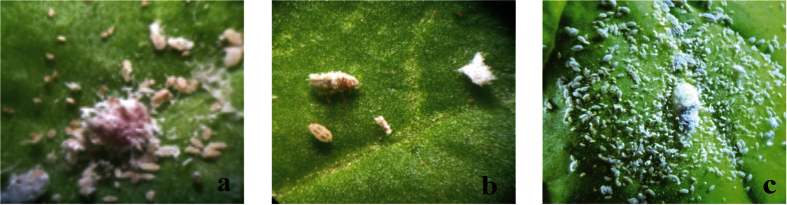
Representative pictures of bioassays performed with *P. solenopsis* feeding on transgenic and control tobacco plants (**a**) Plant expressing Hvt protein (**b**) Plant expressing both HVT and lectin proteins (**c**) Insect reproduction on non-transformed *N. tabacum*.

**Figure 5:**
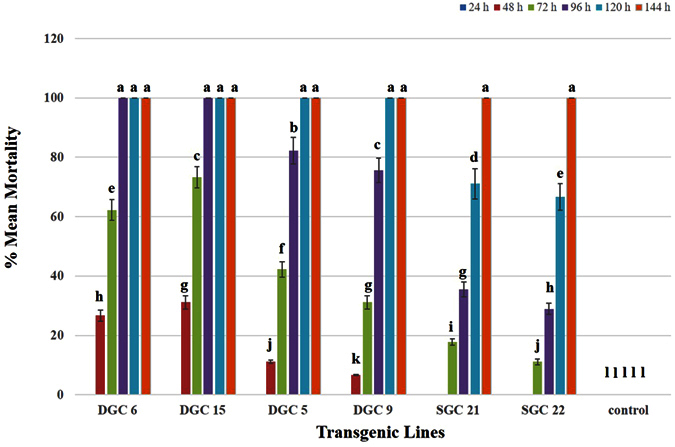
Graph showing mean percent mortality of *P. solenopsis* due to the effects of Hvt (SGC 21 and 22) and combined HVT and lectin toxins (DGC 6, 15, 5, and 9) over time. No insect mortality was observed on non-transgenic control plants. Different letters above the bars indicate P < 0.01, ANOVA followed by Tukey’s HSD test. Each bar represents the mean value of the mortality data collected from five plants during three replicates of the bioassay experiment. The error bars show the standard deviation of the mean values.

Transgenic plants expressing toxins were exposed to *P. solenopsis*, with each plant receiving fifteen *P. solenopsis* individuals. The mortality data of the insects were collected every 24 h and insects were observed for the behavioral changes due to toxin ingestion. During the initial 24 h, insects became adapted to the new environment and settled to feed on transgenic plants. No remarkable changes were observed in the behavior of the insects. The typical effects of the toxin, in particular slower movement of *P. solenopsis*, were observed after 48 h. Almost 28% of the nymphs feeding on DGC 6 and 15 and 10% on DGC 5 and 9 were found dead, but no mortality was recorded on the plants expressing the single gene construct (SGC), plants 21 and 22. After 72 h, 65% of the insects feeding on DGC 6 and 15 were found dead, followed by 35% mortality on DGS 5 and 9. After feeding for 72 h, approximately 15% of the insects were dead on the SGC 21 and 22 plants. After 96 h, almost 100% of the insects were killed on DGC 6 and 15 (Fig. 4b), whereas 80% mortality was recorded on DGC 5 and 9. In the case of the SGC 21 and 22 plants, approximately 38% of the insects were found dead. After 120 h feeding, there was 100% insect mortality on DGC 5 and 9 followed by approximately 77% mortality on SGC 21 and 22 (Fig. 4a). The insects feeding on SGC lines were allowed to feed on plants for another 24 h, at which time 97% of the insects were found dead (Fig. 5). Only a few adult insects were found alive.

The insects feeding on control plants were found to be normal in all aspects. They fed on plants in the usual manner, gained weight, and multiplied enormously. They were normal in their body movements and spread over the whole plant when allowed to feed for longer time period (Fig. 4c).

#### Myzus persicae

**Figure 6:**
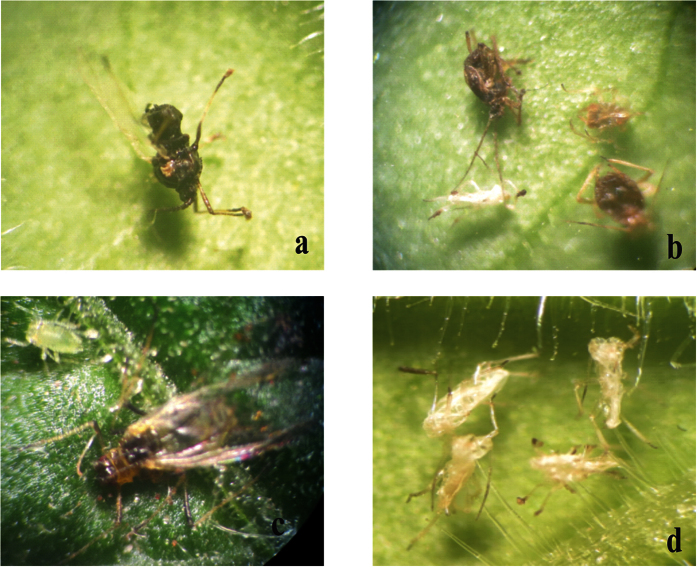
Representative pictures of bioassays performed with *M. persicae* feeding on transgenic tobacco plants (**a**) Plant expressing the Hvt gene (**b**) Plant expressing both Hvt and lectin genes, (**c**) Alive and multiplying *M. persicae* on non-transgenic tobacco (**d**) *M. persicae* exoskeletons indicating growth on non-transgenic tobacco.

**Figure 7:**
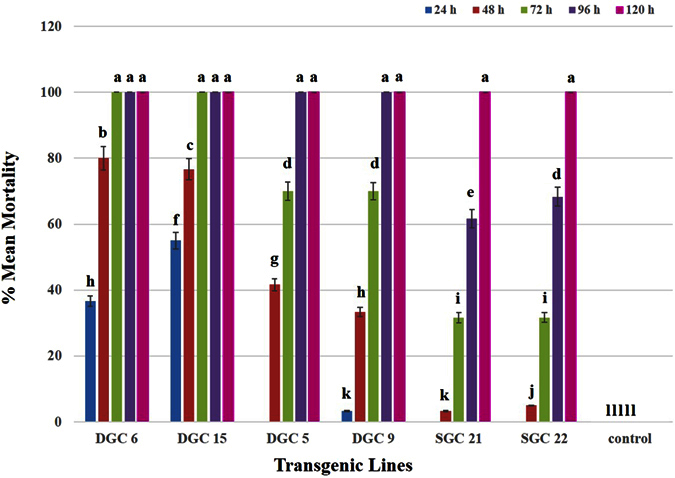
Graph showing mean percent mortality of *M. persicae* due to the effects of Hvt (SGC 21 and 22) and combined HVT and lectin toxins (DGC 6, 15, 5, and 9) over time. No insect mortality was observed on control plants. Different letters above the bars indicate P < 0.01, ANOVA followed by Tukey’s HSD test. Each bar represents the mean value of the mortality data collected from five plants during three replicates of the bioassay experiment. The error bars show the standard deviation of the mean values.

After testing the transgenic plants with *P. solenopsis*, the same plants were exposed to the *M. persicae*, to determine whether there are similar toxic effects. To allow recovery from any possible stress due to insect feeding, plants were left uninfested for 3 to 4 weeks prior to *M. persicae* exposure. After 24 h feeding, behavioral changes, particularly very slow movement, were observed in *M. persicae* feeding on the transgenic plants DGC 5, 9 and SGC 21, 22. In the case of DGC 6 and 15, there was a remarkable difference in the behavior and survival of the insects. Approximately 40% of the individuals, mainly nymphs, were found dead on DGC 6 and 15. None of the nymphs feeding on DGC 6 and 15 survived until the next day. After 48 h feeding, almost 80% mortality was recorded on DGC 6 and 15. Almost 35% of the insects feeding on DGC 5 and 9 were found dead, whereas very low (7–8%) mortality rate were recorded on SGC plants. After 72 h feeding, 100% of the insects were dead on DGC 6 and 15, followed by 70% mortality on DGC 5 and 9 and approximately 40% mortality on SGC 21 and 22. Insects feeding on transgenic plants exhibited the typical effects of toxin uptake, including weight loss, lack of food uptake, lack of movement, jerky movements in the body, and inability to multiply (Fig. 6a,b). On the DGC 5 and 9 lines, 100% of the insects were found dead when they were allowed to feed on for 96 h, followed by almost 70% deaths on SGC 21 and 22. Almost 100% of the insects were dead when allowed to feed on SGC 21 and 22 for another 24 h (Fig. 7). On control plants, *M. persicae* fed normally, gained weight, nymphs moved to the next growth stage, and multiplied rapidly (Fig. 6c). A few exoskeletons were also found on the control plants showing that the insects had molted to the next instar stage (Fig. 6d).

#### Bamisia tabaci

**Figure 8:**
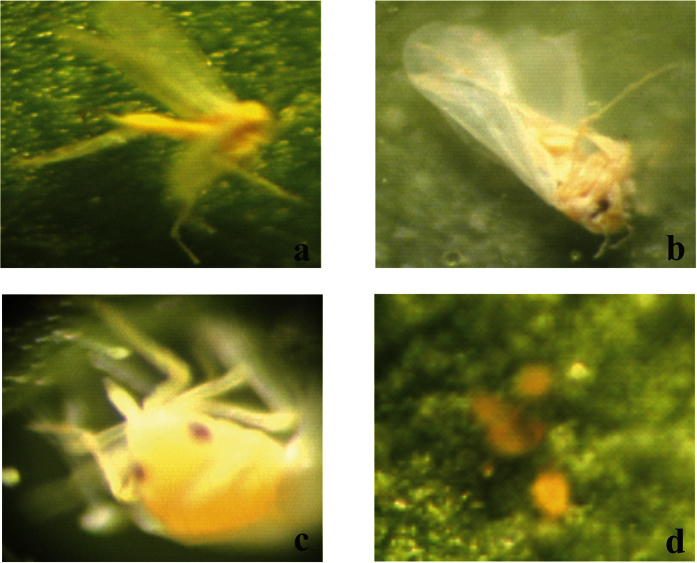
Representative pictures of bioassays performed with *B. tabaci* feeding on transgenic tobacco plants (**a**) Plant expressing the Hvt protein, (**b**) Plant expressing both Hvt and lectin proteins, (**c**) *B. tabaci* feeding on non-transgenic tobacco, (**d**) Eggs laid by *B. tabaci* on non-transgenic tobacco.

**Figure 9:**
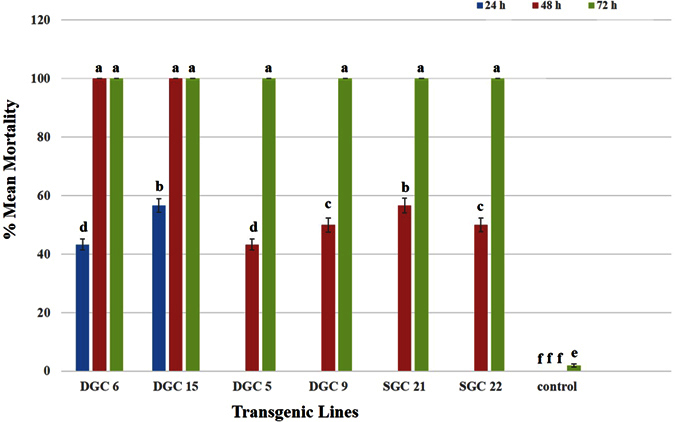
Graph showing mean percent mortality of *B. tabaci* due to the effects of Hvt (SGC 21 and 22) and combined HVT and lectin toxins (DGC 6, 15, 5, and 9) over time. Different letters above the bars indicate P < 0.01, ANOVA followed by Tukey’s HSD test. Each bar represents the mean value of the mortality data collected from five plants during three replicates of the bioassay experiment. The error bars show the standard deviation of the mean values.

Plants were left uninfested for 30–40 days, before being infested with *Bemisia tabaci*. *B. tabaci* took very little time to acclimatize to the plant growth room conditions. After 24 h feeding, almost 50% of the insects feeding on DGC 6 and 15 were found dead. The rest of the insects showed notable behavior changes. In comparison to *P. solenopsis* and *M. persicae*, the toxin effects in *B. tabaci* were quite strong and easily noticed. *B. tabaci* were found with abnormal jerky and shaky movements, abnormal stretching in the body, and other symptoms after 48 h of feeding. There was 100% mortality rate on plants DGC 6 and 15 (Figs 8 and 9) after 48 h, followed by approximately 56% mortality on DGC 5 and 9 and 55% in SGC 21 and 22. The insects were allowed to feed for another 24 h and were found to be dead on all transgenic plants. In case of control plants, the insects remained alive and active, laid eggs, and grew in a regular manner.

All of the tested insects showed similar physical responses to plants expressing Hvt and lectin, including loss of body mass, browning of bodies over time, and stretching of legs inwards in case of DGC or outwards in case SGC, as has been observed previously for neonicotinoid insecticides^62^,^63^. Insects that spent more time on plants also showed some developmental changes as they moved from one growth stage to the next. In case of *M. persicae*, some individuals shed their exoskeletons and moved to the next growth stage, and exoskeletons were found on the plants. *P. solenopsis* stayed on the plants for a longer time, in particular the adult individuals. However, *B. tabaci* did not show such changes, perhaps due to the short time that they spent on the plants.

## Discussion

We have presented results from the expression of two insecticidal proteins in a phloem-specific manner in tobacco. This approach was specifically used to control sap-sucking insects, which take up the toxic proteins when they are feeding. In the case of a constitutive promoter like the CaMV 35S promoter, there is expression in almost all cells of the plant during all developmental stages^64^. Although expression from this promoter is reported to be low in the phloem cells compared to the other plant tissues^64^, expression levels for our GUS constructs with the 35S and phloem-specific BBTV promoters was similar. Expression of transgenes from the 35S promoter can be used to control phloem-feeding insects^64^. However, a potential drawback is that continuous overexpression of insecticidal proteins in multiple tissue types is more likely to cause deleterious effects in the plants.

We used a gene pyramiding technique, both to limit the escape of the insects and for more efficient control. We found significant mortality, and the dead insects showed interesting physical changes. Insects on plants expressing a single protein (Hvt, a neurotoxin) had a low mortality rate when they were examined on a 24-hour basis. Dead insects showed decreased body mass and browning of the bodies, along with their legs being stretched outwards (Figs 4a, 6a and 8a). In contrast, the insects feeding on DGC plants also exhibited a mass decrease and brown bodies, were killed more efficiently, but exhibited different physical characteristics, with their legs folded inward (Figs 4b, 6b and 8b). The differing physical changes of dead insects, in particular the legs being stretched inwards or outwards, are likely due to the effect of the toxins expressed in the host plants. In case of the Hvt, an antagonist of a calcium gating neurotransmitter, the insect nervous system is affected and we observed the stretched insect body^50^,^65^,^66^. In the case of the double protein treatment, a neurotoxin along with a lectin, the lectin acts upon the digestive tract of the insect. That may explain why, along with jerky and shaky movement of the insects, the legs were observed folded inward toward the body. The fecundity of insects was also affected due to feeding on toxin-producing plants^62^,^63^. Insects were observed multiplying on the control plants, but not on the toxin-producing ones. Some of the *B. tabaci* laid eggs on controls, whereas *M. persicae* and *P. solenopsis* were found to develop into the next instars.

In summary, our results show that expression of genes in target tissues and gene pyramiding are efficient techniques for controlling insect pests. Future research can be directed toward the identification of other promoters for tissue-specific expression and targeted control of other insect pest species.

## Methodology

### Promoter identification and cloning

To express the insecticidal genes, phloem-limited promoters were cloned from a Pakistani BBTV isolate^40^. Two viral components, the capsid protein (CP) and the nuclear shuttle protein (NSP) were chosen for amplifying the promoters. The intergenic region (IR) was considered as the promoter and sequence-specific primers (Table 1 in supplementary data) were used to amplify the whole IR by PCR. The deleted part of the NSP intergenic region was used as reported^45^. The PCR reaction mixture was prepared using the Thermo Scientific, USA *Taq* polymerase kit, containing 1X reaction buffer, MgCl_2_, 2 mM dNTPs, 10 pmol of each primer, 10 ng of template and 0.5 units of *Taq* polymerase. The PCR reaction was run with the following temperature profile: initial denaturation temperature 94 °C for 5 min, 1 cycle; followed by 35 cycles of 94 °C for 30 sec, 50 °C for 30 sec, 72 °C for 45 sec; and final extension at 72 °C for 10 min. *Sac*I and *Hind*III restriction sites were used for cloning into the pJIT166 expression vector^67^, replacing the 2X 35S promoter upstream of the GUS reporter gene. In the next step, the complete expression cassette from pJIT166 was excised and cloned into the pGreen0029 binary vector^68^ at restriction sites *Sac*I and *Xho*I, which makes the construct ready for further cloning of genes under this promoter.

#### Agrobacterium transformation

One μg of the cloned and confirmed plasmid was transformed into *Agrobacterium tumefaciens* strain GV3101 by electroporation^69^. Transformed cells carrying the construct were selected on LB agar plates containing antibiotics (rifampicin 25 μg/mL, tetracycline 10 μg/mL and kanamycin 50 μg/mL). Different colonies were picked and the presence of the promoter was confirmed by PCR. A single-colony culture was prepared from a PCR-positive colony in 50 mL LB containing antibiotics (rifampicin 25 μg/mL, tetracycline 10 μg/mL and kanamycin 50 μg/mL) in a 250 mL conical flask. The medium was incubated at 28 °C and 1.8 RCF (relative centrifugal force) for 48 h. After 48 h, the culture was pelleted by centrifugation in a 50 mL Falcon tube. The pellet was resuspended in 10 mM MgCl_2_, 20 μM acetosyringone was added, and the sample was kept overnight at 4 °C. *Nicotiana tabacum* (tobacco) plants were infiltrated with the activated culture and kept under controlled conditions of 28 ± 2 °C and 16L/8D for 4–5 days.

#### Histochemical staining assay

GUS (β-glucuronidase) was used as a reporter gene to check promoter efficiency. Five days post infection (dpi) leaves were detached and vacuum infiltrated with 50 mM sodium phosphate buffer pH 7.0, containing ethylene-diaminetetraacetic acid (EDTA), Triton-X100 and X-Gluc (a substrate for β-glucuronidase)^70^. Leaves were kept in buffer overnight and incubated at 37 °C. Afterwards the incubation buffer was discarded and leaves were bleached with 100% ethanol until all the chlorophyll was removed and blue GUS staining was clearly visible. GUS expression in leaves was quantified by using ImageJ 1.5 software^71^,^72^. The leaf area showing GUS expression was selected and subjected for the quantification.

### Insecticidal gene constructs

The Hvt (GenBank accession number AJ938032) gene was amplified by PCR using gene specific primers (Table S2 in the supplementary data) and cloned under the NSP promoter of BBTV in the pGreen0029 vector, using restriction sites *Hind*III and *Xba*I.

For double gene constructs, two individual cassettes were synthesized by Bio Basic Inc., Canada. One cassette contained the NSP promoter, Hvt, and the CaMV terminator, whereas the other consisted of the CP promoter, lectin (GenBank accession number DQ255944), and the CaMV terminator. Both cassettes were cloned in pGreen0029 using restriction sites *Sac*I and *Xho*I.

### Plant transformation

The final construct in the binary vector pGreen0029 was transformed into *Agrobacterium tumefaciens* strain GV3101 by electroporation. A single colony culture, after confirmation by PCR, was prepared in 50 mL LB containing antibiotics (50 μg/mL kanamycin, 10 μg/mL tetracycline, and 25 μg/mL rifampicin) at 28 °C and 1.8 RCF for 48 h. *Nicotiana tabacum* was transformed with the leaf disc method, using a modified version of a published protocol^73^. For selecting transformants, MS medium was supplemented with 50 mg/L kanamycin, 250 mg/L cefotaxime, 250 μg/L 1-napthalene acetic acid (NAA) and 1 mg/L 6-benzylaminopurine (BAP). The putative transformants were shifted to soil for further analysis.

### Transgene Analysis

Seeds collected from T_0_ plants were germinated on kanamycin so all of them have at least one copy of transgene. These plants were not tested for homozygosity. DNA was extracted from fresh leaves of transgenic plants at the T_1_ stage using the cetyltrimethylammonium bromide (C-TAB) method^74^ and confirmed for the presence of toxin genes and promoters by PCR using sequence specific primers and also by sequencing.

### RNA extraction and cDNA synthesis

Fresh samples were taken from transgenic plants after washing with diethylpyrocarbonate (DEPC) and kept in liquid nitrogen. Total RNA from transgenic plants at the T_1_ stage was extracted by using Plant RNA Purification Reagent (Invitrogen catalog number 12322-012) following a standard published protocol^75^. Freshly extracted RNA was quantified using a Nanodrop (Thermo Scientific, USA) and was used to synthesize cDNA with the cDNA Synthesis Kit (Thermo Scientific, USA). Two μg of total RNA was used as a template for the synthesis of cDNA.

### Real-time PCR for transgene analysis

Real-time PCR or quantitative PCR (qPCR) was performed to check the relative expression of transgenes. Here, SYBER Green technology was used to study the gene expression in transgenic plants. All of the optimization and experiments were carried out using iQ^TM^5 iCycler (BioRad) equipment. The 18S ribosomal RNA gene was used as a reference gene and its expression was compared with the expression of transgenes. Finally, the six best lines were selected for analysis by qPCR, four from double gene construct (DGC) transgenes and two from single gene construct (SGC) or Hvt transgenes. Three plants from each line were selected for gene expression analysis.

The reaction mixture (25 μL) consisted of 12.5 μL of 1X iQ SYBER Green Supermix, 50 ng of sample cDNA, and gene specific primers (Table S2 in the supplementary data). The PCR reactions were carried out in a 96-well optical plate in an iQ^TM^5 iCycler (BioRad) and all of the samples were run in triplicate. PCR was carried out using the profile: 1 cycle at 94 °C for 5 min followed by 40 cycles, each consisting of 94 °C for 30 sec, 52 °C for 30 sec and 72 °C for 45 sec. The melt curve analysis was started from 52 °C, with an increase in temperature of 0.5 °C/min.

### Insect rearing

Three sucking pests, *P. solenopsis*, *M. persicae* and *B. tabaci* were used to check the effect of toxic proteins. Insects were collected from the field and were allowed to feed and multiply on cotton plants in a glass house under controlled conditions of 37 ± 2 °C, 16L/8D and ≈60% humidity. The insects settled very well on cotton and multiplied enormously.

### Insect bioassays

Transgenic plants were grown in a growth room under controlled conditions of 26 ± 2 °C and 16L/8D. After confirmation by PCR, 8 lines of the DGC and 5 lines of the SGC were grown under controlled conditions in the T_1_ stage. Four lines of the DGC and two lines of the SGC with the strongest transgene expression were used for insect bioassays. Five plants of each line at the 3–4 leaf stage were used for bioassays. Different phloem feeding insects infested on transgenic plants under controlled conditions. Fifteen individuals of *P. solenopsis*, 20 individuals of *M. persicae* and 20 of *B. tabaci* of mixed populations, consisting of both nymphs and adults, were allowed to feed on plants to check the effects of toxic proteins. Equal numbers of control plants (wild type *N. tabacum*) with equal numbers of insects were also used to get comparable mortality data. The percent mortality was calculated by the following formula: (number of insect pests found dead on the plant/(number of the pests allowed to feed on the plant − number of insects that absconded)) × 100.

### Statistical Analysis

ANOVA and Tukey’s SD test were used to assess significance in the mortality data using the statistical software program SPSS-20^76^.

## Additional Information

**How to cite this article**: Javaid, S. *et al*. A transgenic approach to control hemipteran insects by expressing insecticidal genes under phloem-specific promoters. *Sci. Rep*. **6**, 34706; doi: 10.1038/srep34706 (2016).

## Supplementary Material

Supplementary Information
