# Supplementary material for: A transgenic approach to control hemipteran insects by expressing insecticidal genes under phloem-specific promoters
Source: Sci Rep. 2016 Oct 6;6:34706. doi: 10.1038/srep34706 (PMC5052614; doi:10.1038/srep34706)
Supplement: Supplementary Information [file srep34706-s1.doc]

# A transgenic approach to control hemipteran insects by expressing insecticidal genes under phloem-specific promoters

**Shaista Javaid**^1, 2, 3^, **Imran Amin**^1^, **Georg Jander**^3^, **Zahid Mukhtar**^1^, **Nasir A. Saeed**^1^ and **Shahid Mansoor**^1*^

^1^Agricultural Biotechnology Division, National Institute for Biotechnology and Genetic Engineering (NIBGE), P. O. Box 577, Jhang Road, Faisalabad, Pakistan

^2^Pakistan Institute of Engineering and Applied Sciences (PIEAS), Nilore, Islamabad, Pakistan

^3^Boyce Thompson Institute for Plant Research, 533 Tower Road, Ithaca, NY 14853, USA

***Corresponding author: Shahid Mansoor**

Agriculture Biotechnology Division, National Institute for Biotechnology and Genetic Engineering, Jhang Road, Faisalabad, Punjab, Pakistan.

Ph. # +92-41-2651471

Cell # +92-300-7944841

Fax # +92-41-2651472

[shahidmansoor7@gmail.com](mailto:shahidmansoor7@gmail.com)

**Email addresses of all the authors**

Shaista Javaid

[shaistajavaid85@gmail.com](mailto:shaistajavaid85@gmail.com)

Imran Amin

[imranamin1@yahoo.com](mailto:imranamin1@yahoo.com)

Georg Jander

[gj32@cornell.edu](mailto:gj32@cornell.edu)

Zahid Mukhtar

zahidmukhtar@yahoo.com

Nasir A. Saeed

nasaeed@nibge.org

Shahid Mansoor

[shahidmansoor7@gmail.com](mailto:shahidmansoor7@gmail.com)

**Promoter analysis**

The sequences of the NSP and CP proposed promoters were analyzed by PlantCARE to identify cis-regulatory elements. All of the important motifs, e.g. A-Box, G-Box, G-box, GC-motif, CAAT-Box and TATA- Box were found in the proposed promoter region (Figure S1 & S2). All of these elements are necessary for a sequence to act as promoter.


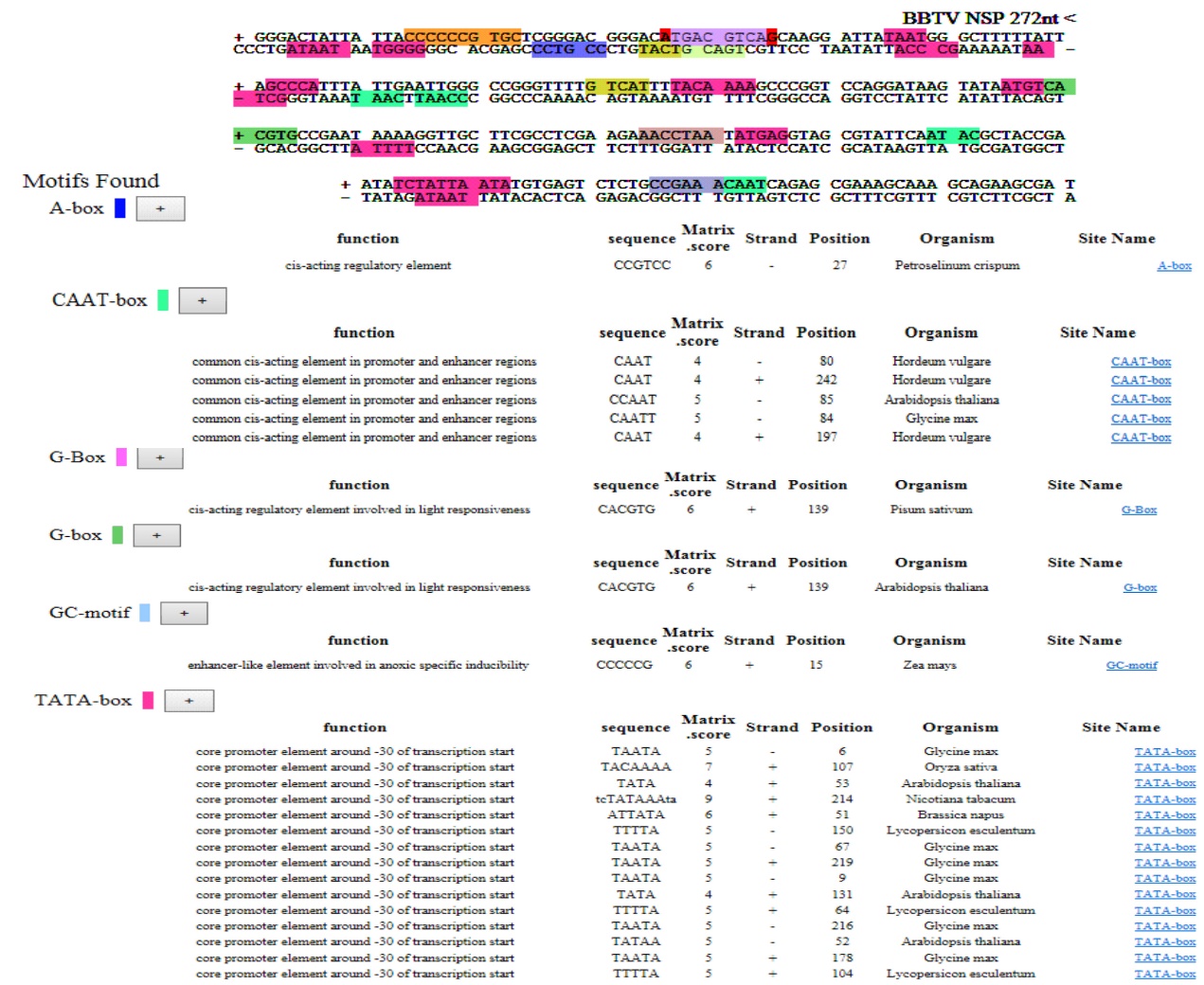


**Figure S1.** Cis-regulatory element analysis for the NSP promoter sequence using PlantCARE


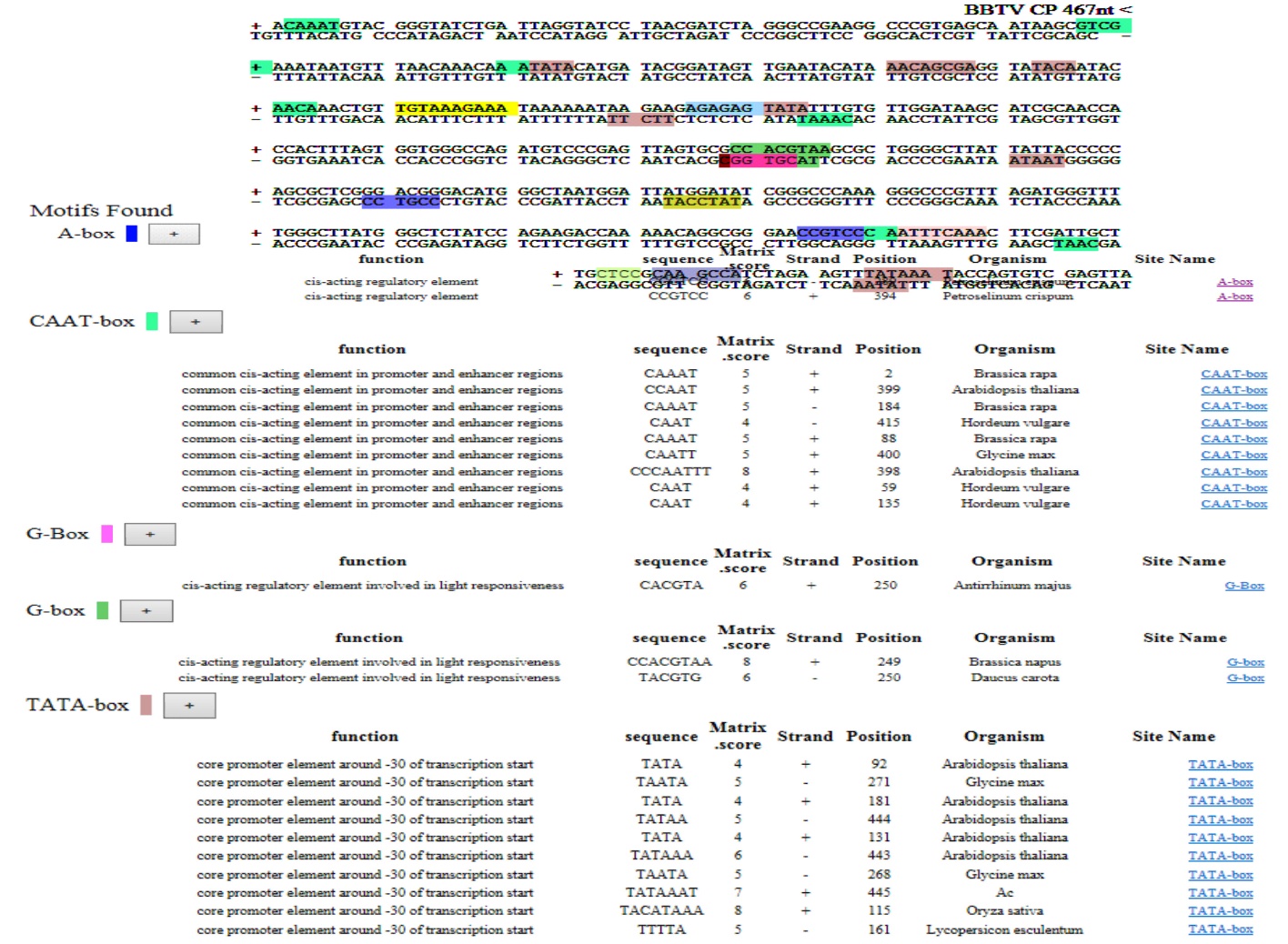


**Figure S2.** Cis-regulatory elements analysis for the CP promoter sequence using PlantCARE

**Mode of action of lectin in insect gut**


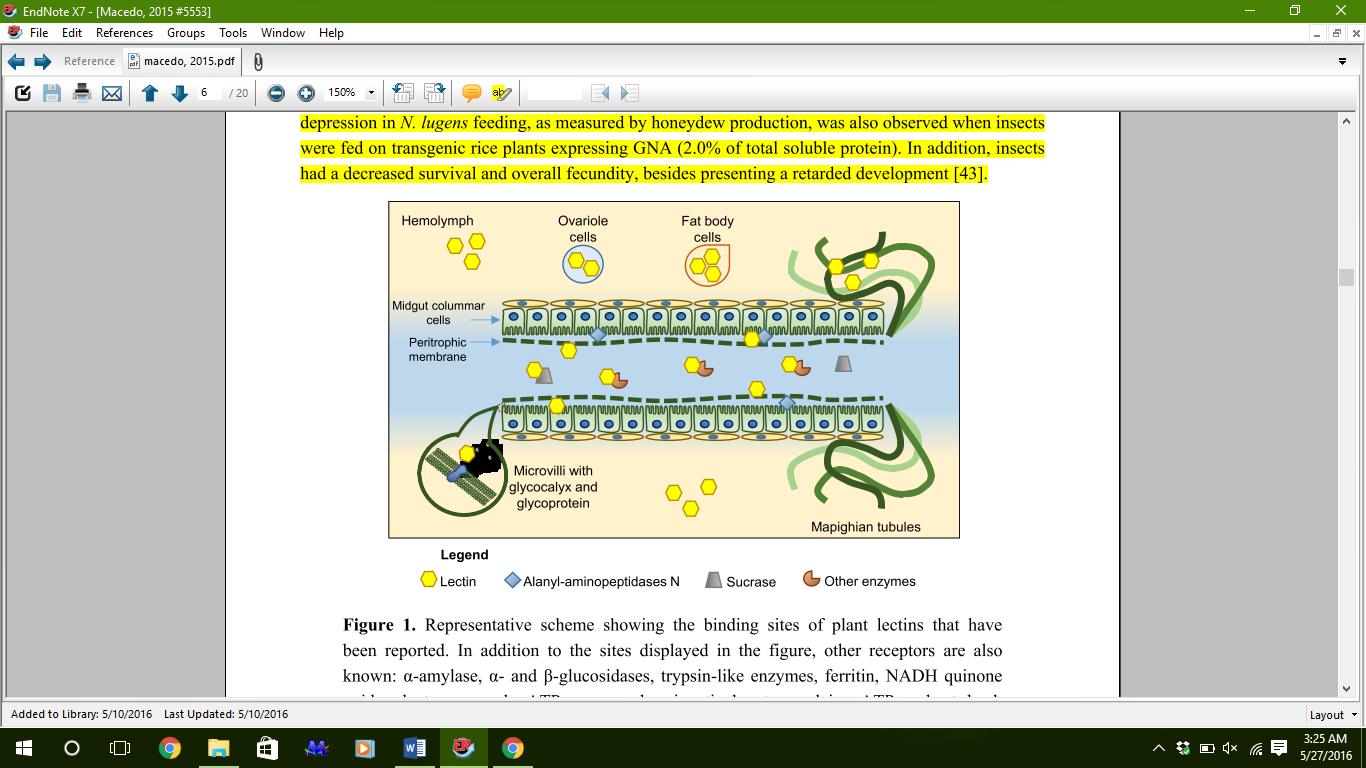


**Figure S3.** Representative scheme showing the binding sites of plant lectins that have

been reported. In addition to the sites displayed in the figure, other receptors are also

known: α-amylase, α- and β-glucosidases, trypsin-like enzymes, ferritin, NADH quinone

oxidoreductase, vacuolar ATPase; sarcoplasmic reticulum type calcium ATPase, heat shock

protein 70, β-subunit ATP synthase and Clathrin heavy chain^1^


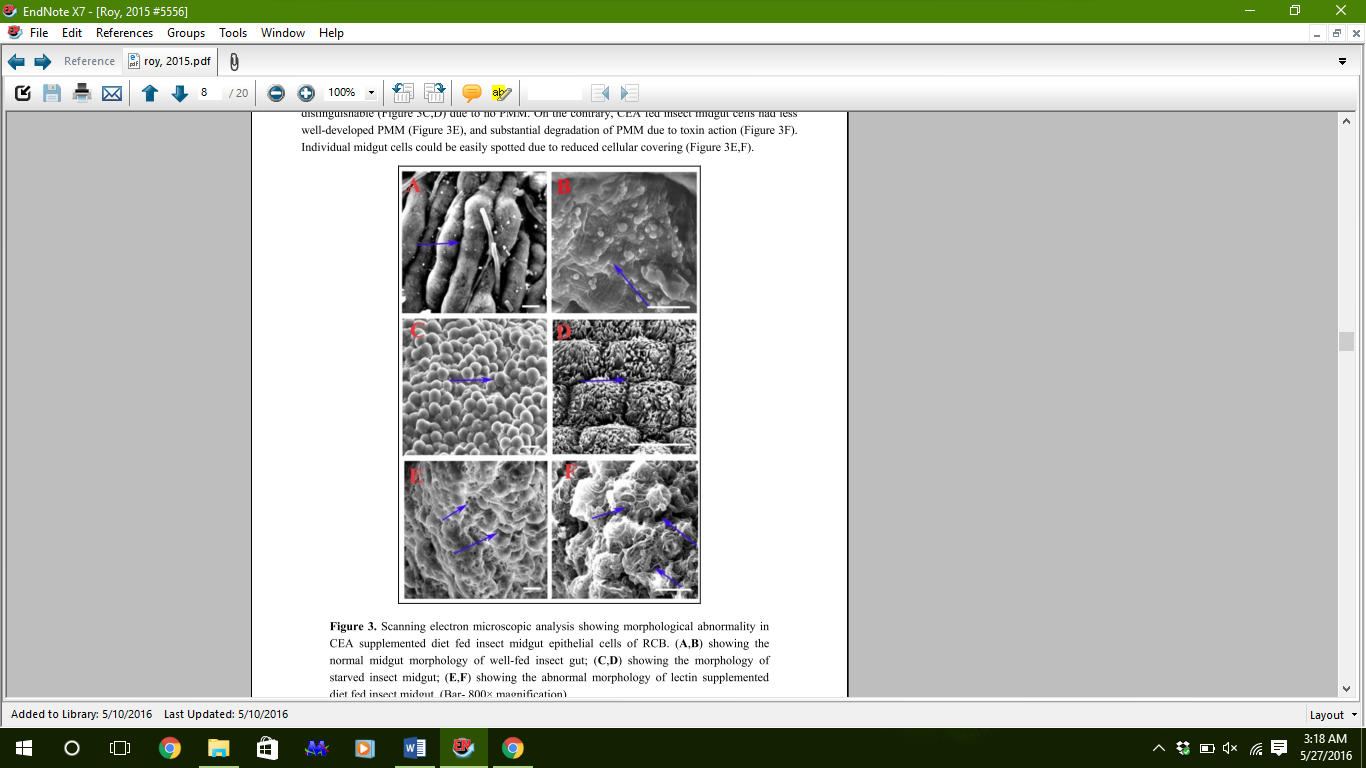


**Figure S4.** Scanning electron microscopic analysis showing morphological abnormality in CEA supplemented diet fed insect midgut epithelial cells of red cotton bug (RCB). (A,B) showing the normal midgut morphology of well-fed insect gut; (C,D) showing the morphology of starved insect midgut; (E,F) showing the abnormal morphology of lectin supplemented diet fed insect midgut. (Bar- 800× magnification)^2^

**Mode of Action for Hvt**

**
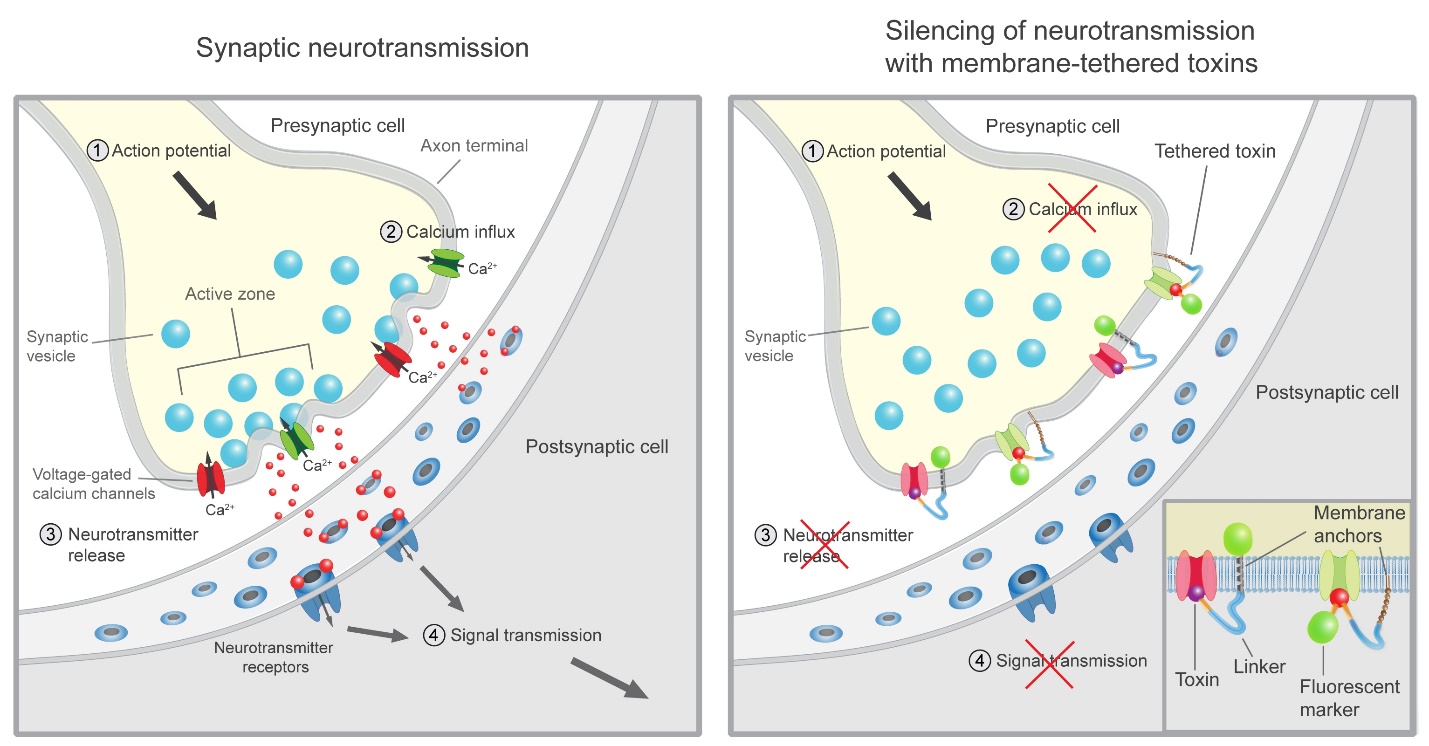
**

**Figure S5.** This is proposed model for Hvt mode of action; being an antagonist of Ca^2+^ channel how Hvt blocks the verve impulse^3^

**Melt-Curve for qPCR**

**
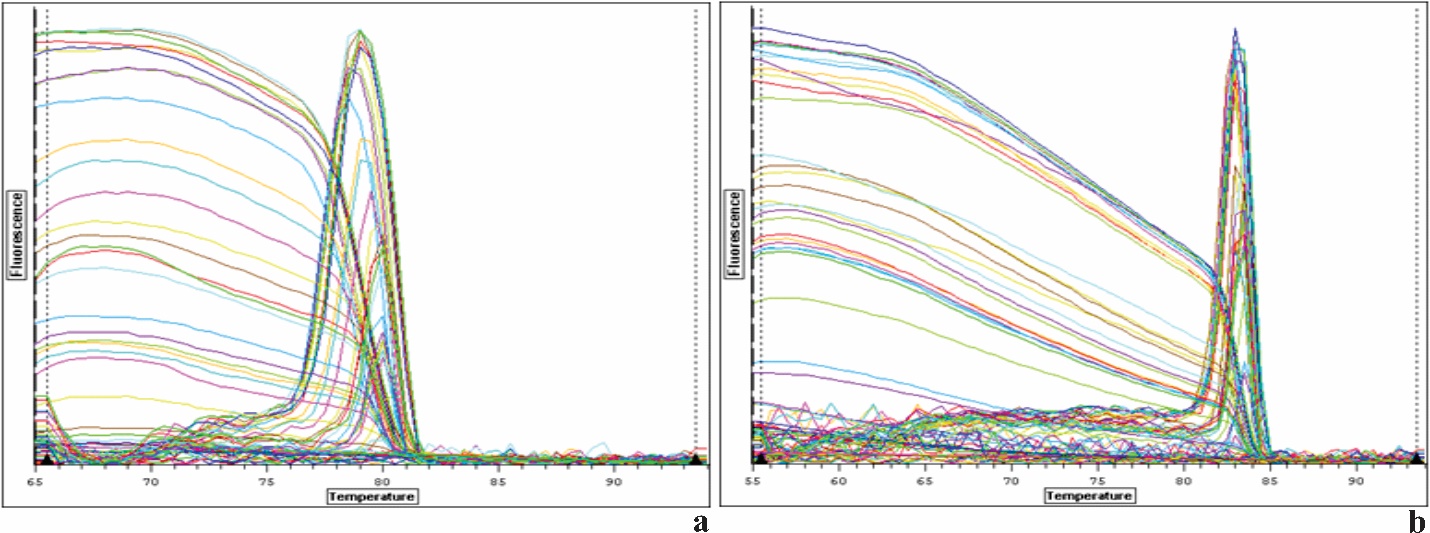
**

**Figure S6.** Melt curve analysis for qPCR expressing single peak indicates there is no non-specific amplification during qPCR (a) Melt curve for Hvt (b) Melt curve for lectin

## **Primers sequences used for amplification**

| **Primer Name** | **Primer Sequence** |
| --- | --- |
| NSP Forward | 5’-ATGAGCTCGTATACTAATCTCTGATTGG-‘3 |
| NSP Reverse | 5’-ACAAGCTTCATCGCTTCTGCTTTGCTTT-‘3 |
| CP Forward | 5’-CAGAGCTCTATGTTTATGTAAACATAAA-‘3 |
| CP Reverse | 5’-ACAAGCTTCTAACTCGACACTGGTATTT-‘3 |

##### **Table 1.** Specific primers used for promoter amplification

| **Primer Name** | **Primer Sequence** |
| --- | --- |
| Hvt Forward | 5’-CG AAGCTTATGTCACCAACTTGCATACC-‘3 |
| Hvt Reverse | 5’-ACTCTAGATTAATCGCATCTTTTTACGG-‘3 |
| Lectin Forward | 5’-CCAAGCTTATGGCCAGGAACCTACTGAC-‘3 |
| Lectin Reverse | 5’-ACTCTAGATTAGTAGGTCCAGTAGAACC-‘3 |

##### **Table 2.** Specific primers for insecticidal genes amplification

**References**

1 Macedo, M. L. R., Oliveira, C. F. & Oliveira, C. T. Insecticidal activity of plant lectins and potential application in crop protection. *Molecules* **20**, 2014-2033 (2015).

2 Roy, A. & Das, S. Molecular mechanism underlying the entomotoxic effect of colocasia esculenta tuber agglutinin against dysdercus cingulatus. *Insects* **6**, 827-846 (2015).

3 Auer, S. *et al.* Silencing neurotransmission with membrane-tethered toxins. *Nature Methods* **7**, 229-236 (2010).
